# Active metabolites and potential mechanisms of *Notopterygium incisum* against obstructive sleep apnea Syndrome (OSAS): network analysis and experimental assessment

**DOI:** 10.3389/fphar.2023.1185100

**Published:** 2023-08-31

**Authors:** Peijun Liu, Weihua Tang, Dong Zhao, Pan Zhou, Ke Hu

**Affiliations:** ^1^ Department of Respiratory and Critical Care Medicine, Renmin Hospital of Wuhan University, Wuhan, China; ^2^ Department of Respiratory and Critical Care Medicine, The Central Hospital of Enshi Tujia and Miao Autonomous Prefecture, Enshi, China; ^3^ Department of Radiology, The Central Hospital of Enshi Tujia and Miao Autonomous Prefecture, Enshi, China

**Keywords:** OSAS, intermittent hypoxia, medicinal plants, *Notopterygium incisum*, *Hansenia weberbaueriana*, the botanical drug, PTGS2

## Abstract

**Background:** *Notopterygium incisum* K.C. Ting ex H.T. Chang, a synonym of *Hansenia weberbaueriana* (Fedde ex H. Wolff) Pimenov & Kljuykov, is an anti-inflammatory medicinal plant. Although abrnotopterol has been reported to be its primary active metabolite, the other metabolites and their mechanisms of action remain unclear. This study aims to investigate the potential mechanisms by which its active metabolites treat Obstructive Sleep Apnea Syndrome (OSAS) through network analysis and experimental assessment.

**Methods:** The metabolites and potential targets of *Notopterygium incisum* were extracted from public databases. We searched for OSAS-related genes in the Genecards, OMIM, PharmGkb, TTD, and DrugBank databases. Cytoscape 3.9.0 was used to construct the drug-target-disease network and screen for hub genes. Human bronchial epithelial (HBE) cells were cultivated in normoxia and chronic intermittent hypoxia (CIH) medium for 24 h. Interleukin-6 (IL-6), tumor necrosis factor-alpha (TNF-α), and prostaglandin E2 (PGE2) were quantified using enzyme-linked immunosorbent assay (ELISA). Prostaglandin-endoperoxide synthase 2(PTGS2) mRNA was detected using RT-qPCR, while PTGS2 and nuclear factor-kappa B (NF-κB) proteins were identified using Western blot analysis. Co-Immunoprecipitation (CoIP) and Western blotting were utilized to evaluate the ubiquitination of PTGS2 in HBE cells.

**Results:** Pterostilbene and notopterol, isolated from *Notopterygium incisum*, had potential therapeutic effects on OSAS. The PTGS2 and estrogen receptor alpha (ESR1) hub genes were associated with OSAS. The pathway enrichment analysis focuses on the NF-κB, apoptosis, and HIF-1A pathways. In response to CIH, pterostilbene and notopterol decreased IL-6, TNF-α, and PGE2 levels. The NF-κB pathway was activated by an increase in PTGS2 levels. Pterostilbene promoted proteasome-mediated ubiquitination of PTGS2 protein and reduced PTGS2 levels, inhibiting the NF-κB pathway.

**Conclusion:** This study reveals the active metabolites of *Notopterygium incisum* and hub genes involved in treating OSAS, which provide a basis for the follow-up development and exploitation of the botanical drug.

## Introduction

Obstructive sleep apnea syndrome (OSAS) is a common respiratory disease (Agha and Johal, 2017). The main clinical characteristics are repeated complete or partial upper airway collapse during sleep, and the pathophysiological mechanism is chronic intermittent hypoxia (CIH) (Matsumura et al., 2019). It can induce daytime lethargy, poor mental state, insomnia, and other clinical symptoms due to long-term intermittent hypoxia (Stradling, 2009). Drowsiness caused by OSAS can increase the accident rate and endanger public safety (Peker et al., 2021). OSAS can aggravate nervous and circulatory system diseases, increasing the risk of atrial fibrillation in patients with coronary heart disease (Peker et al., 2022). Currently, nocturnal non-invasive ventilator therapy is the most common treatment strategy, and continuous positive airway pressure (CPAP) is the most widely accepted treatment strategy (Lao et al., 2022).

Drugs such as Provigil and Ozawade are both used to treat OSAS (Avellar et al., 2016). In a trial of 50 OSAS patients treated with CPAP, Y. Inoue observed that all patients experienced significant daytime drowsiness, but even oral Provigil did not completely alleviate the symptoms (Inoue et al., 2016). Ozawade significantly decreases sleepiness and ameliorates disease severity in OSAS patients with good CPAP compliance (Zaccara et al., 2021). The European Medicines Agency authorized it as a therapy for OSAS-related excessive daytime sleepiness (Kollb-Sielecka et al., 2017). However, these medications vary significantly between populations and are ineffective for some individuals (Avellar et al., 2016). The primary pathophysiological mechanism underlying OSAS is CIH, which is tightly connected to systemic inflammation (Ding et al., 2014). Numerous systemic inflammatory pathways, including interleukin-6 (IL-6), nuclear factor-kappa B (NF-κB), tumor necrosis factor-alpha (TNF-α), and P38 mitogen-activated protein kinase-dependent pathways, are dependent on oxidative stress (Wu et al., 2013). IL-6 serves as a pivotal cytokine implicated in orchestrating inflammation and immune responses. NF-κB modulates genes intertwined with inflammation and immunity, while TNF-α enhances inflammatory conditions by its involvement in immune responses, cellular apoptosis, and the regulation of inflammatory disorders. These molecules intricately interact within a complex network, governing the body’s inflammatory processes (Akhtar et al., 2020). Specific medicinal plants, such as essential oils, have potent antioxidant, anti-inflammatory, and antimicrobial activities (Risaliti et al., 2019). As a result, we also investigated the impact of botanical drugs on OSAS.

*Notopterygium incisum* K.C. Ting ex H.T. Chang, a synonym of *Hansenia weberbaueriana* (Fedde ex H. Wolff) Pimenov & Kljuykov, yields a traditional Chinese botanical drug growing well in Gansu Province, Tibet, and other high-altitude regions, and is composed of many useful monomers (Wu et al., 2010). It was first documented in Shennong Ben Cao Jing, a seminal work in ancient Chinese botanical drugs. Characterized by a bitter, aromatic, and slightly spicy flavor in Traditional Chinese Medicine, it exhibits distinct properties that contribute to therapeutic applications, strategically employed to alleviate conditions such as wind-cold, damp-heat, and inflammation by explicitly targeting the pathways of the bladder and kidney meridians (Azietaku et al., 2017). Notopterol, its primary metabolite, has been proven to treat rheumatoid arthritis by targeting the JAK-Stat signaling system, hypoxia-induced pulmonary hypertension, and COVID-19 (Wang et al., 2020b; Nazari-Khanamiri and Ghasemnejad-Berenji, 2022). CIH can increase pulmonary hypertension, although notopterol can decrease symptoms (Huang et al., 2022). *Notopterygium incisum* may have an impact on inhibiting oxidative stress and systemic inflammation induced by hypoxia.

Consequently, we speculate that the active metabolites of *Notopterygium incisum* can influence the mechanism of CIH induced by OSAS. Network analysis and molecular biology offer promising approaches for analysing active metabolites and disease targets, thereby facilitating the discovery of botanical drug metabolites for effective disease treatment (Li and Zhang, 2013). However, it is essential to acknowledge that network analysis may generate false positives. To address this potential concern, we have complemented our approach with experimental investigations to provide a more robust and validated understanding of the underlying mechanisms.

## Materials and methods

### Extraction of pharmaceutical ingredients

A quantity of dried rhizomes of *Notopterygium incisum* (100 g) was crushed at 15,000 r/min for 5 min using a high-speed shear mixer (T25-digital, IKA, Germany) to obtain a fine powder. The powder was mixed with 4 L of water and soaked for 2 h. After sonication for 20 min, the mixture was transferred to a three-necked flask fitted with a condensing reflux unit and heated at 100°C for 6 h. After separating and removing solid residues, the extract was concentrated to 0.8 L using a rotary evaporator (RV-8 V, IKA, Germany). The concentrated solution was cooled at two to sixºC for 10 h, and cold methanol was added to precipitate the insoluble residues. The mixture was then centrifuged using an ST 16R centrifuge (Thermo Fisher Scientific, Massachusetts, United States) and filtered through a 0.22 µm membrane to obtain samples. The samples were stored at 4°C in the dark.

### Metabolites prediction

TCMSP (https://tcmspw.com/index.php) was used to collect the metabolites of *Notopterygium incisum* based on a drug-like property value of 0.10. Furthermore, the UniProt network platform (https://www.uniport.org) incorporated the target proteins, which were converted to gene symbols by Perl software (https://www.perl.com).

### Identification of OSAS disease targets

The databases Genecards (http://www.genecards.org/), OMIM (https://omim.org/), PharmGkb (https://www.pharmgkb.org/), and TTD (http://db.idrblab.net/ttd) were searched using the terms “OSAS”, “sleep apnea syndrome”, and “sleep apnea”. The Venn program in R was used to cross-reference metabolite potential targets with disease genes to find potential “disease targets.”

### Screening of disease-related metabolites and hub genes

Cytoscape 3.9.0 was used to construct a network of disease-related metabolites and potential disease-target genes, with the size of the nodes changed based on the interaction relationship. The potential therapeutic targets of *Notopterygium incisum* for OSAS were imported into the STRING database (http://string-db.org/), and the data were entered into Cytoscape software (version 3.9.0) to compute the center degree and intermediary center (Tang et al., 2015).

### Gene function and pathway enrichment analysis

Gene Ontology (GO) and Kyoto Encyclopedia of Genes and Genomes (KEGG) pathway analyses were performed using the “clusterprofile” and “pathview” packages in R software version 3.6.2 to investigate potential therapeutic target genes for OSAS. In bubble and bar charts, the *p*-adjust represents importance, associated with red.

### High-performance liquid chromatography (HPLC)

The concentrations of notopterol (nol) and pterostilbene (pte) in the test sample solution were determined by HPLC. The equipment utilized included the following: LC-2030C high-performance liquid chromatography system (Shimadzu), Inertsil ODS-3 column (4.6 mm × 250 mm, 5 µm), mobile phase: acetonitrile +0.5% glacial acetic acid (50:50), a flow rate of 1.0 mL/min, detection wavelength of 305 nm, injection volume of 10 μL, and column temperature of 35 °C. We utilized isocratic elution and Labsolutions software for HPLC to collect data and generate spectra.

### Cell culture and CIH model

Human bronchial epithelial (HBE) cells were donated by the Department of Thoracic Surgery, Renmin Hospital of Wuhan University. HBE cells were cultured in RPMI-1640 medium containing 10% fetal bovine serum and 1% penicillin/streptomycin. The humidified incubator contained 5% CO_2_, and the temperature was 37°C. The CIH model was established in a hypoxic incubator at 5% O_2_ for 5 min, followed by 21% O_2_ for 10 min for 24 h (Azietaku et al., 2017). HBE cells were intermittently hypoxic for 24 h, followed by the addition of 10 mM cycloheximide (C112766, Aladdin, Shanghai, China), and the expression levels of prostaglandin-endoperoxide synthase 2 (PTGS2) protein were analysed by Western blotting at 3, 6, and 9 h, respectively.

### Cell proliferation assay CCK8

HBE cells in logarithmic growth were equally dispersed in 96-well plates with different medication dosages of RPMI-1640 complete medium solution. The cells were subjected to diverse concentration gradients of pterostilbene (P108000, Aladdin, Shanghai, China) or notopterol (N418583, Aladdin, Shanghai, China) for varying time periods (24 h, 48 h), aiming to explore their effects on cell proliferation. The cells were harvested after 24 and 48 h. After adding 10 µL of CCK8 solution to each well, the cells were maintained in the dark for 2 hours. The plate absorbance (450 nm, dual-wavelength) was evaluated with a microplate reader (EnSight, PerkinElmer, United States), cell viability was calculated, and the CCK-8 assays were repeated three times.

### Cell viability assay and trypan blue staining

After treating the cells with different concentration gradients of pterostilbene or notopterol for varying durations, HBE cells were washed twice with PBS and stained with 0.4% trypan blue staining solution for 5 min. It was then washed twice with saline and imaged under a light microscope. Trypsin-EDTA solution was used to digest the cells and collect them. Following centrifugation, 100 μL of the cell suspension was transferred into a 0.5 mL centrifuge tube. Subsequently, 100 μL of Trypan blue stain (0.4%) was added, gently mixed and allowed to stand for 3 min. Cell counting was performed using a hemocytometer. The cell survival rate was calculated using the following formula: cell survival rate = (total number of cells - number of blue cells)/total number of cells × 100%, and the trypan blue staining assay was repeated three times.

### Experimental procedures

The indicated mass of compounds was accurately weighed and dissolved in 10 mL of DMSO, respectively: 51.26 mg of pterostilbene (P108000, Aladdin, Shanghai, China) and 106.323 mg of notopterol (N418583, Aladdin, Shanghai, China). After meticulous filtration through a 0.22 μm sterile syringe filter, solutions were prepared as storage concentrations of pterostilbene (20 mM) and notopterol (30 mM). The stock solutions were stored at −80°C in the dark.

After the HBE cells reached 90% confluence, the complete RPMI-1640 medium containing the following specified components was replaced separately 6 h ahead of time: 1)0.1% DMSO as a solvent control; 2) 0.1% 20 mM DMSO-solubilized pterostilbene storage solution (final concentration of 20 μM); and 3) 0.1% 30 mM DMSO-solubilized notopterol storage solution (final concentration of 30 μM). Subsequently, three of these groups were incubated for 24 h under normoxia conditions, recorded as Con, Con-pte, and Con-nol. In addition, the other three groups were exposed to CIH conditions for 24 h, and the three groups were recorded as CIH group, CIH-pte group, and CIH-nol group. Finally, cell supernatants and cells were collected separately to obtain cell supernatants and cell lysates for ELISA, ROS, and Western blot analysis, respectively. The difference for immunofluorescence-based ROS detection experiments is that the cells need to be inoculated in advance in a 24-well plate with a coverslip placed at the bottom.

### ELISA analysis

The HBE cell culture medium was collected and centrifuged for 5 min at 3,000 rpm. The supernatant was centrifuged and assayed according to the manufacturer’s instructions for the glutathione (GSH) kits (A006-2-1, Jiancheng Bioengineering Institute, Nanjing, China). The levels of IL-6, TNF-α, and prostaglandin E2 (PGE2) were measured according to the manufacturer’s kit instructions (E-EL-H6156, H0109c,0034c, Elabscience, Wuhan China), and the cell assays were repeated three times.

### Reactive oxygen species (ROS) measurements

ROS production was detected by *in situ* labeling of HBE cells with dihydroethidium, a ROS fluorescent probe (1:500, R353922, Aladdin, Shanghai, China). HBE cells were incubated at 37°C for 30 min with DHE staining solution (10 µM) and counterstained with DAPI solution. Specimens were examined using a fluorescence microscope (IX71, Olympus, Japan). ROS-positive cells labeled with fluorescein will emit a red light (excitation 510 nm, emission 610 nm), whereas DAPI (2 μg/mL, G1012, Wuhan, Servicebio)-labeled nuclei emit a blue light (excitation 340 nm, emission 488 nm). A minimum of three distinct regions were selected for each sample. Fluorescence intensities were measured with ImageJ (National Institutes of Health, Bethesda, MD).

### Western blot analysis

Total protein was obtained by lysing HBE cells with RIPA buffer containing 1% PMSF and the protease inhibitor cocktail, and total protein was determined by the BCA method. The samples were transferred to PVDF membranes. The PVDF membranes were blocked for 2 h with 10% skim milk. The primary antibodies were incubated overnight at 4°C, and the primary antibodies used as follows, GAPDH (1:1000, GB15002, Servicebio, Wuhan, China), β-actin (1:1000, GB15003, Servicebio), NF-kB (1:1000, GB11997, Servicebio),PTGS2 (1:2000,27308-1-AP, Proteintech, Wuhan, China), LaminB (1:2000, GB111802, Servicebio), and Ubiquitin (1: 500, AG3164, Beyotime, Shanghai, China). The membrane was then incubated for 2 h with secondary antibodies. Immunoreactive bands were displayed and visualized with an image capture system (ChemiDoc MP, Bio-Rad).

### RT‒qPCR analysis

After intermittent hypoxia treatment, RT-qPCR was used to detect the mRNA of PTGS2 in HBE cells, and GAPDH was used as an endogenous reference. According to the manufacturer’s instructions, total RNA was isolated from HBE cells using the RNA Isolation Kit (9108, Takara). The mRNA expression of PTGS2 was determined using the One Step TB Green PrimeScript RT‒PCR kit (RR096A, TaKaRa, Japan). Real-time PCR analysis was performed using SYBR Master Mix on a Bio-Rad Connect Real-Time PCR platform (Bio-Rad, United States). Thermal cycling conditions: 1. Reverse transcription reaction, 42°C for 5 min, 95°C for 10 s 2. PCR, 95°C for 5 s, 60°C for 30 s, repeat: 40 times. The RT‒qPCR primer sequences are listed in Table 1. Target gene expression was determined using the 2^−ΔΔCT^ method (Livak and Schmittgen, 2001).

**TABLE 1:** The primers for RT‒qPCR.

Target	Forward primer (5′-3′)	Reverse primer (3′-5′)
PTGS2(Human)	GAA​AAC​TGC​TCA​ACA​CCG​GAA	GCA​CTG​TGT​TTG​GAG​TGG​GT
GAPDH(Human)	CTT​TGG​TAT​CGT​GGA​AGG​ACT​C	GTA​GAG​GCA​GGG​ATG​ATG​TTC​T

### Co-Immunoprecipitation (CoIP)

HBE cells were separated into three groups: control (normoxia), chronic intermittent hypoxia (CIH), and chronic intermittent hypoxia plus pterostilbene (CIH-pte). Total protein was extracted using a CoIP lysis solution (20 mM Tris-HCl, pH 7.5, 150 mM NaCl, 1% Triton X-100), and 800 µg of total protein from each sample was added to PTGS2 antibody (1:2000,27308-1-AP, Proteintech, China) for specific immunosorbent, followed by Protein G Mag Sepharose kit (GE Healthcare, Bucking-Hamshire, UK) for centrifugal co-precipitation. The coprecipitated products were washed and denatured by boiling in sample loading buffer at 100°C and centrifuged. After the supernatant was removed, Western blotting was performed to detect PTGS2 protein expression, and IgG-IP was used as a negative control.

### Statistical analysis

The experimental data were analysed with GraphPad Prism 8.0, and the experimental results are expressed as the mean ± standard deviation (X ± S). The result was tested with the Shapiro‒Wilk normality and the variance homogeneity test. One-way analysis of variance (ANOVA) was used to calculate differences between multiple groups, followed by Tukey’s *post hoc* test. Statistically significant differences were defined as *p* < 0.05.

## Results

### Prediction of disease-related metabolites of notopterygium incisum

**FIGURE 1:**
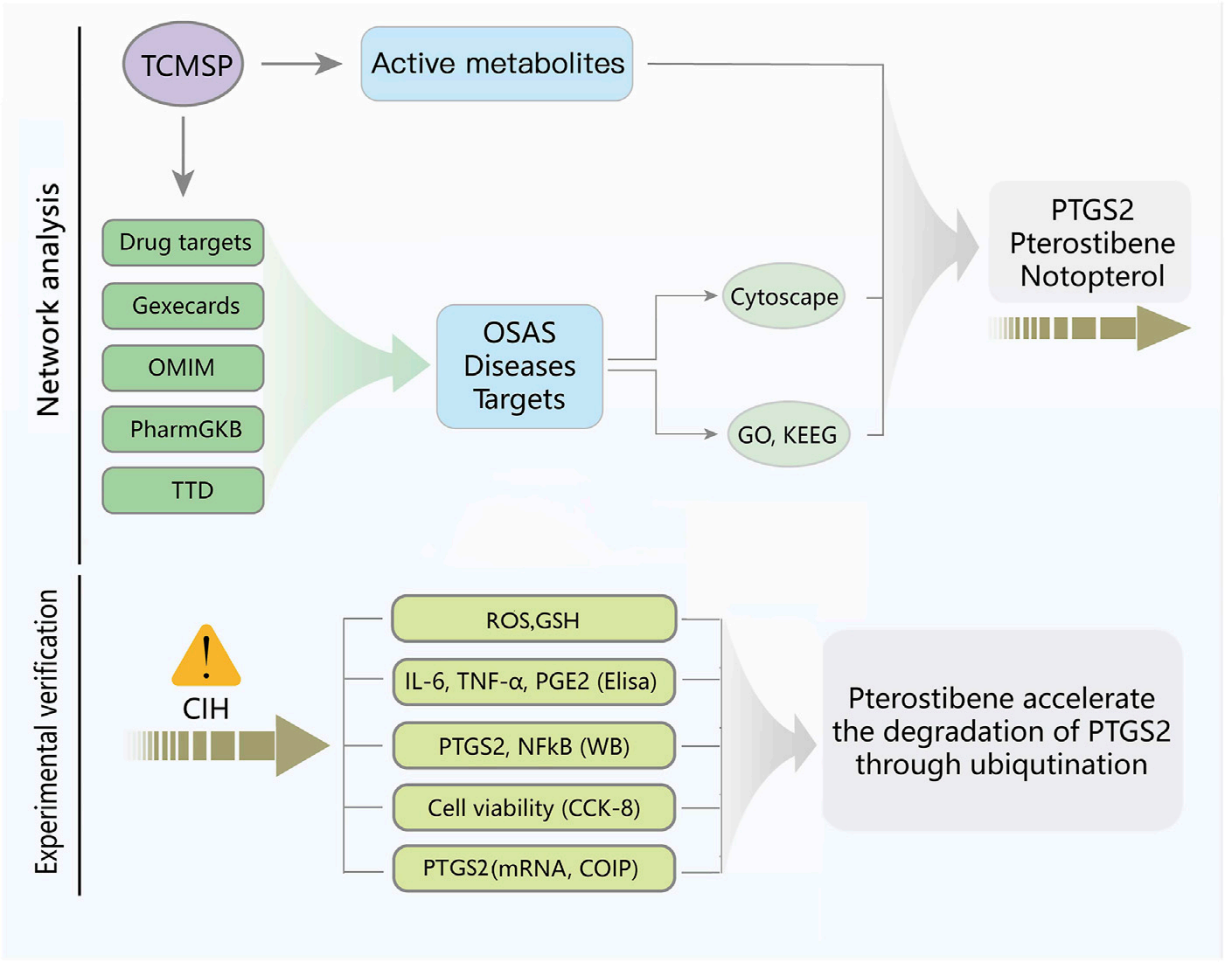
Flowchart of the investigation.

**FIGURE 2:**
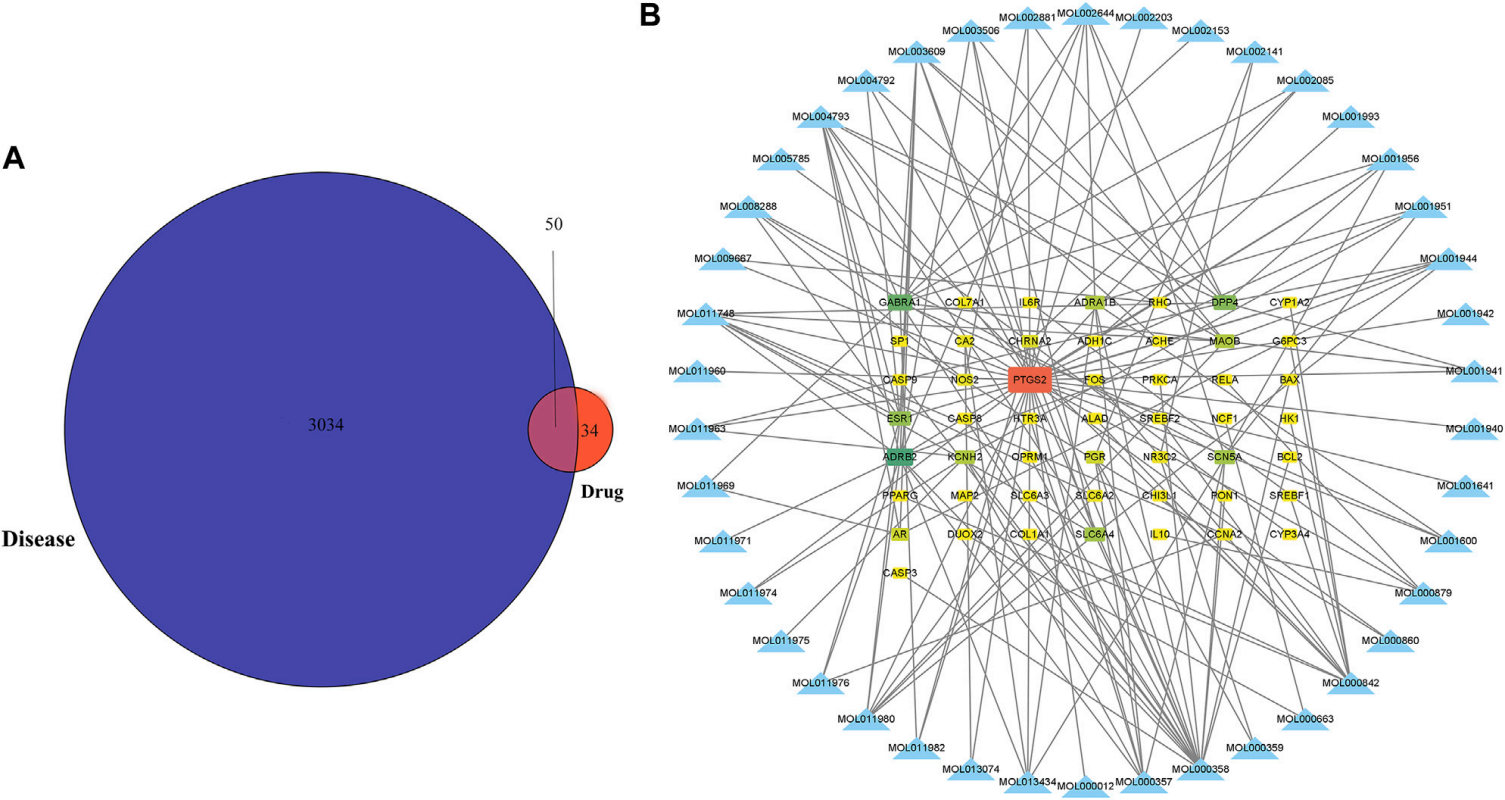
The metabolites of *Notopterygium incisum* can alleviate OSAS by acting on target genes. **(A)** Genes of pharmacological and disease targets that overlap. **(B)** Construction of a network between the metabolites and OSAS disease genes, with 92 nodes and 157 edges. Abbreviation: OSAS, Obstructive Sleep Apnea Syndrome.

Figure 1 depicts the flowchart for the ongoing investigation. The TCMSP database contained 73 metabolites of *Notopterygium incisum* with drug-likeness>0.1, 42 of which had target proteins. Finally, 84 target proteins corresponding to 42 metabolites were obtained through the Unipro-KB database. A total of 3084 OSAS-related genes were searched in the GeneCards, OMIM, PharmGkb, TTD, and DrugBank databases. We intersected the two datasets to obtain 50 target genes (Figure 2A). Cytoscape software established the network between the disease-related metabolites and the targets, with 92 nodes and 157 edges (Figure 2B).

### PPI network analysis of target genes

**FIGURE 3:**
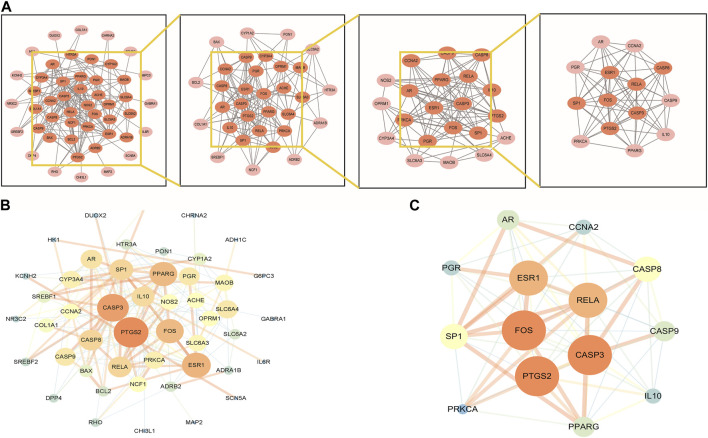
PPI network analysis and clustering analysis of the disease targets. **(A)** PPI network of possible OSAS therapeutic targets of *Notopterygium incisum*. **(B)** The size and color of the nodes are positively correlated with the PPI network Betweenness and Degree values. **(C)** The significant proteins were identified from the PPI network based on betweenness and degree values. Abbreviation: PPI, protein-protein interaction.

**FIGURE 4:**
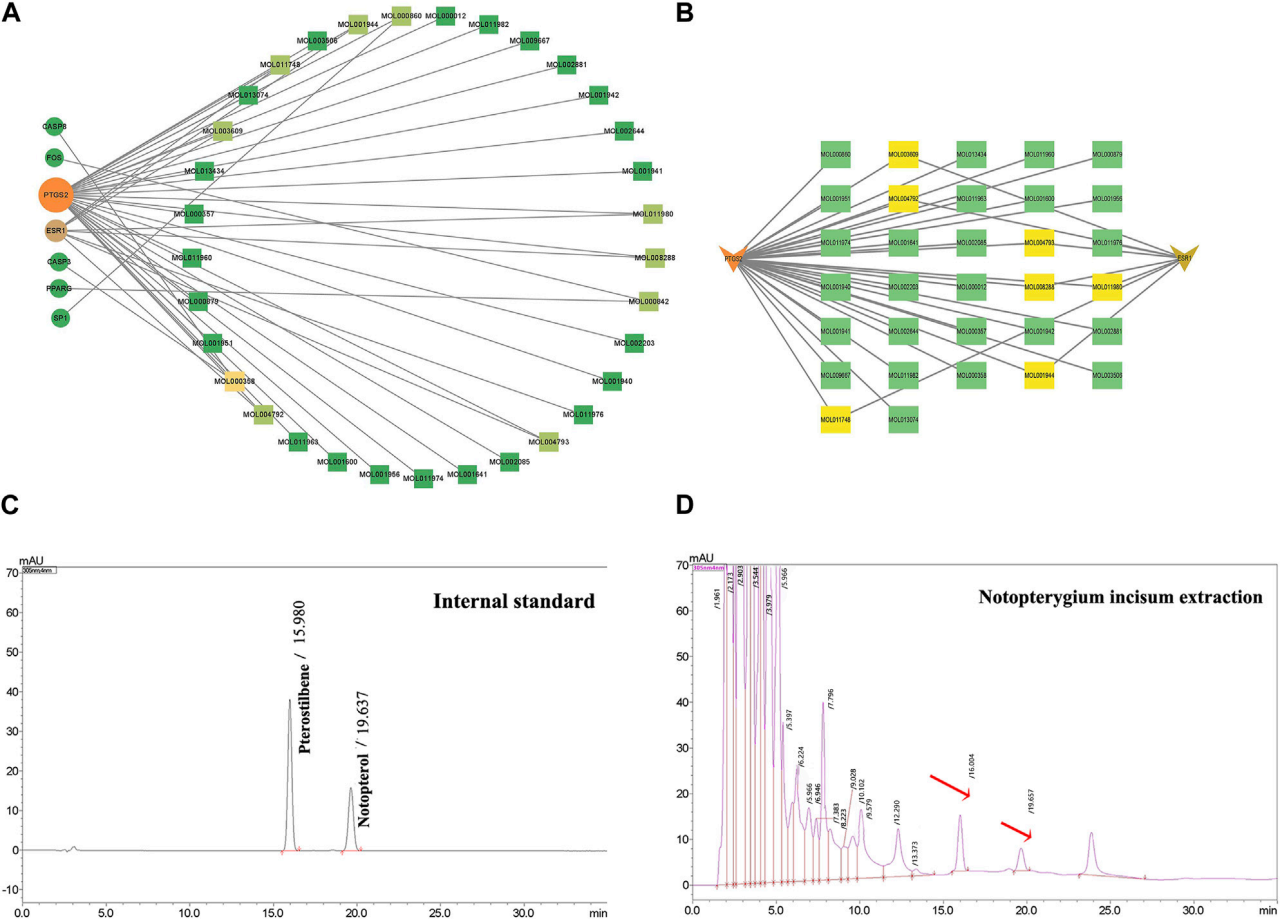
PTGS2 and ESR1 are primary OSAS-related targets. **(A)** Network interactions between 7 hub genes and 33 of the most important disease-related metabolites. **(B)** PTGS2 or ESR1 has a network interaction with their respective metabolites. **(C, D)** Pterostilbene and notopterol were observed in *Notopterygium incisum* by HPLC. Abbreviations:PTGS2,prostaglandin-endoperoxide synthase 2; ESR1, estrogen receptor alpha.

To explore the interaction relationship among 50 target genes, we processed them through the PPI network, which contained 48 nodes and 181 edges. Two isolated nodes were not involved in the PPI network. The median degree of the network was 15.08, and the average betweenness was 65.54. Further analysis revealed that the subnetwork had a mean betweenness of 4.57 and a mean degree of 16.86 (Figures 3A,B). The hub genes in the network were PTSG2, FOS, and CASP3 (Figure 3C). Cytoscape 3.9.0 was used to investigate the interaction between the 7 hub genes and the 33 metabolites (Figures 4A,B), including beta-sitosterol, pterostilbene, and 10 other metabolites that acted on more than 2 target genes. We discovered that pterostilbene has the greatest oral bioavailability. OSAS can aggravate oxidative stress and affect a variety of organs. Pterostilbene is the replacement for resveratrol, which has an antioxidative stress role in numerous disorders (Teng et al., 2021). Notopterol is the primary metabolite of *Notopterygium incisum*, which has anti-inflammatory and antioxidant properties (Chen et al., 2021a). As a result, we selected pterostilbene and notopterol for vitro cell validation experiments. Pterostilbene and notopterol were discovered in *Notopterygium incisum* after being confirmed using the HPLC (Figures 4C,D).

### GO enrichment analysis

**FIGURE 5:**
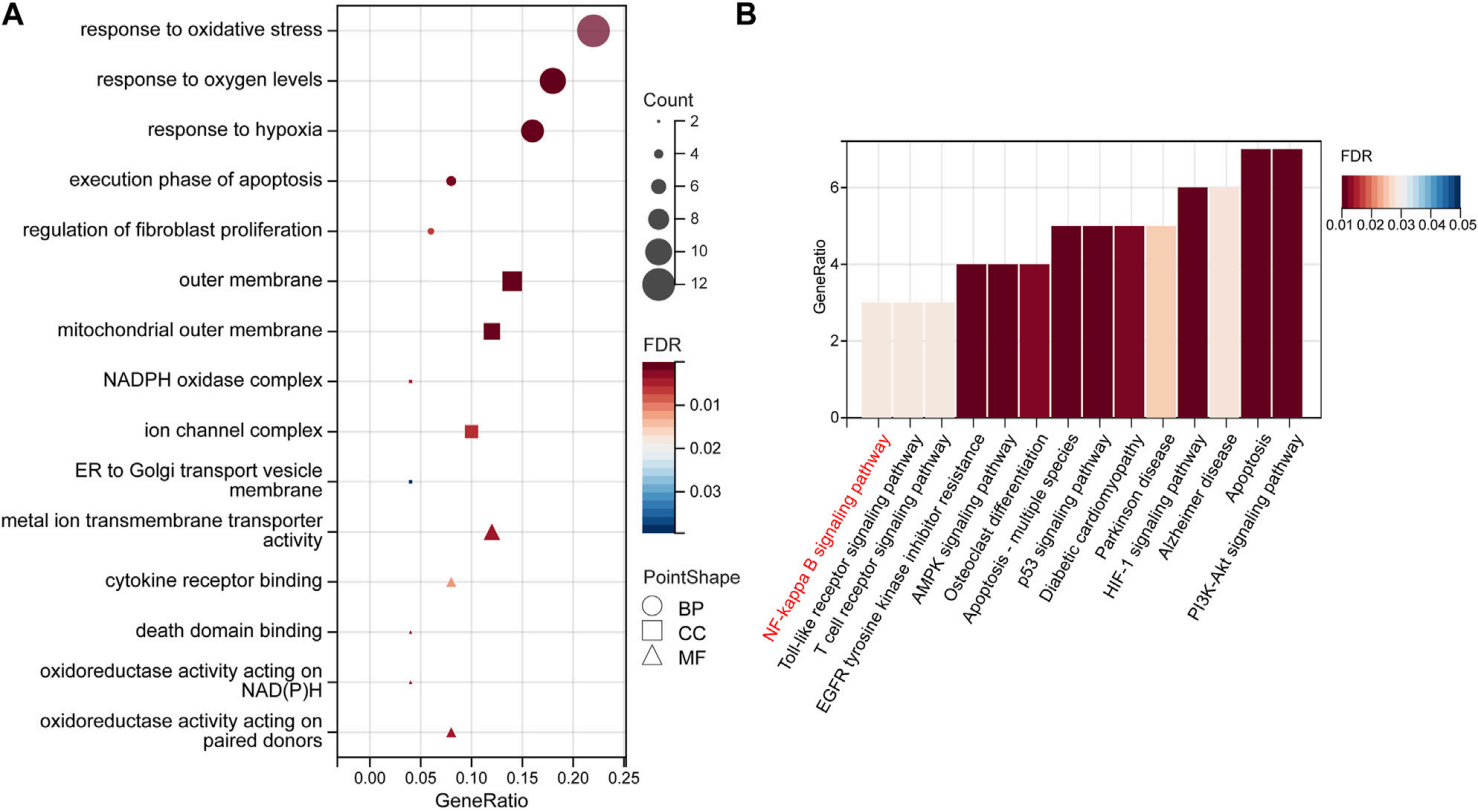
GO enrichment and KEGG pathway analyses. **(A)** The top 15 GO enrichment terms. GO terms include biological process (BP), subcellular localization (CC), and molecular function (MF). **(B)** The top 14 signaling pathways according to the KEGG database. Abbreviations: GO, Gene Ontology; KEGG, Kyoto Encyclopedia of Genes and Genomes.

GO and KEGG analyses were performed to identify the enriched pathways of the 50 target genes. According to our findings, the majority of biological processes (BP) involve reactions to “oxidative stress,” “oxygen content,” and “hypoxia.” Cellular Component (CC) mainly includes the “outer membrane,” “mitochondrial,” “outer membrane,” and “NADPH oxidase complex.” “Metal-ion transmembrane transporter activity,” “cytokine receptor binding,” and “oxidoreductase activity” are the most important molecular functions (MF) (Figure 5A, Supplementary Table S1).

### KEGG enrichment analysis

A total of 39 KEGG-enriched pathways were obtained (Figure 5B), with 10 signaling pathways conforming to *p* < 0.05, including “apoptosis,” “HIF-1”, “PI3K-Akt”, “NF-kappaB” (Supplementary Table S2). The hub gene, PTSG2, is related to the NF-κB and Alzheimer’s disease pathways, whereas RELA and BCL2 are associated with the NF-κB pathway. In our subsequent experiments, it was predicted that pterostilbene and notopterol could affect NF-κB pathway activity through PTGS2.

### Pterostilbene and notopterol reduce CIH-induced cell death

**FIGURE 6:**
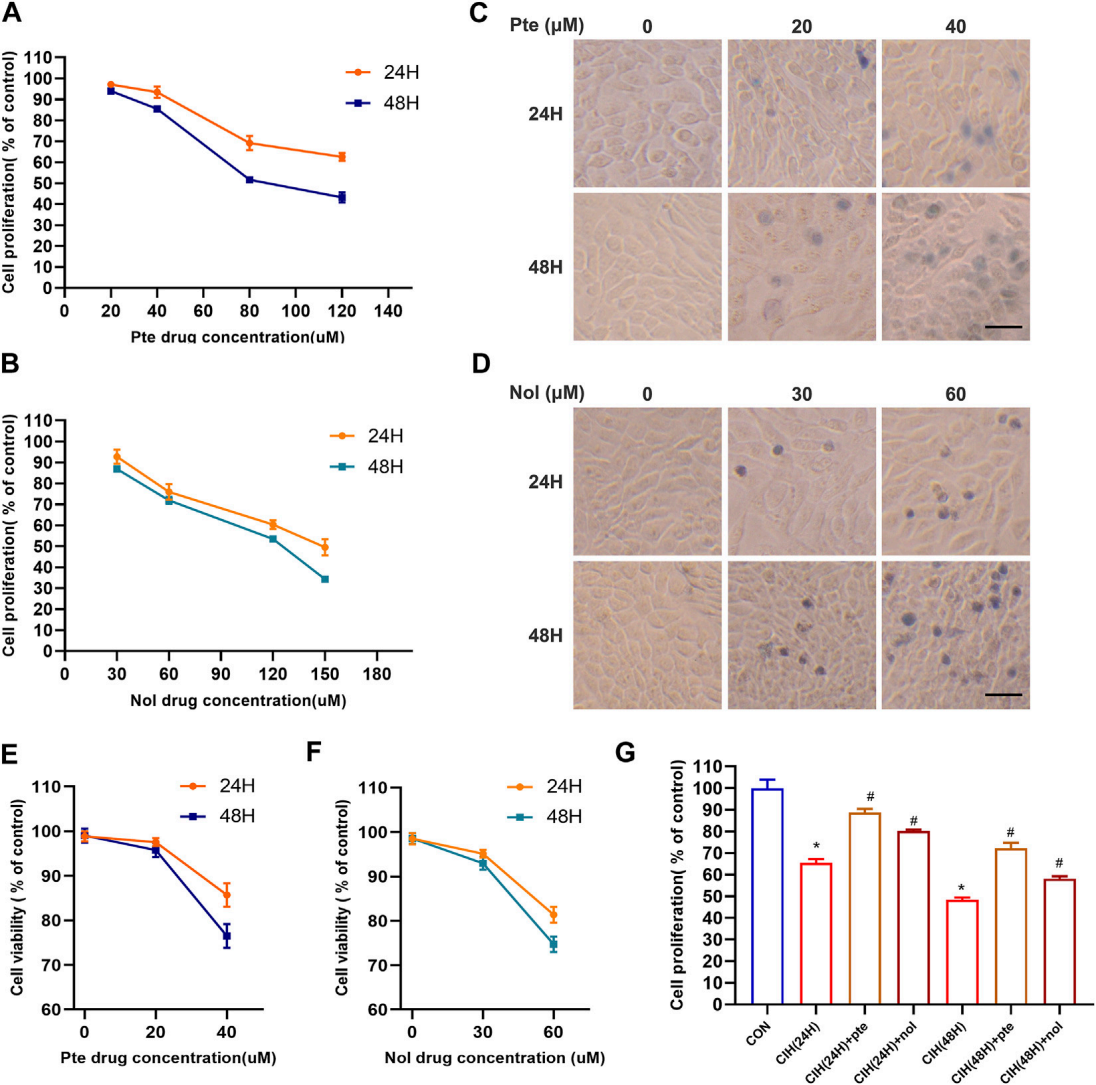
Pterostilbene and notopterol decrease cell death caused by CIH. **(A)** Alterations in the viability of HBE cells result from being treated with different doses of pte for 24 and 48 h **(B)** The viability of HBE cells treated with nol was altered after 24 and 48 h **(C, D)** Trypan blue staining images of cells were captured under a light microscope following treatment with a time/concentration gradient of pte or nol (400×, bar scale = 50 μm). **(E) (F)** Cell viability changes were observed following treatment with a time/concentration gradient of pte or nol. **(G)** Cell activity test in the CIH cell model following early addition of pte and nol.*p < 0.05 compared to the Con group,^#^ *p <* 0.05 compared to the CIH group. Abbreviations: CIH, chronic intermittent hypoxia; HBE, human bronchial epithelial; Pte, pterostilbene; Nol, notopterol.

To evaluate whether pterostilbene and notopterol mitigate the damage in the CIH model, after treatment with different concentrations of pterostilbene for 24 and 48 h, the viability of HBE cells was significantly inhibited following treatment with a high concentration (80 µM) of pterostilbene (Figure 6A). High concentration notopterol (90 µM) suppressed HBE cells viability after 24 and 48 h (Figure 6B). Trypan blue staining revealed that 20 μM pterostilbene had minimal toxicity, with >90% cell survival; at 40 μM pterostilbene, cell survival fell below 90%/80% after 24/48 h (Figures 6C,E). At 30 μM notopterol, cell toxicity was low, with survival >90%; at 60 μM, cell survival dropped to 80%/75% after 24/48 h (Figures 6D,F). Therefore, 20 μM pterostilbene and 30 μM notopterol are acceptable concentrations. Cell viability was lower in a CIH environment for 24 and 48 h than in the normal oxygen group; adding 20 μM pterostilbene and 30 μM notopterol significantly reduced cell death (Figure 6G). Pterostilbene and notopterol have been shown to reduce cell damage, and the specific mechanism has been explored in the following phase.

### Pterostilbene and notopterol reduce the oxidative stress and inflammation caused by CIH

**FIGURE 7:**
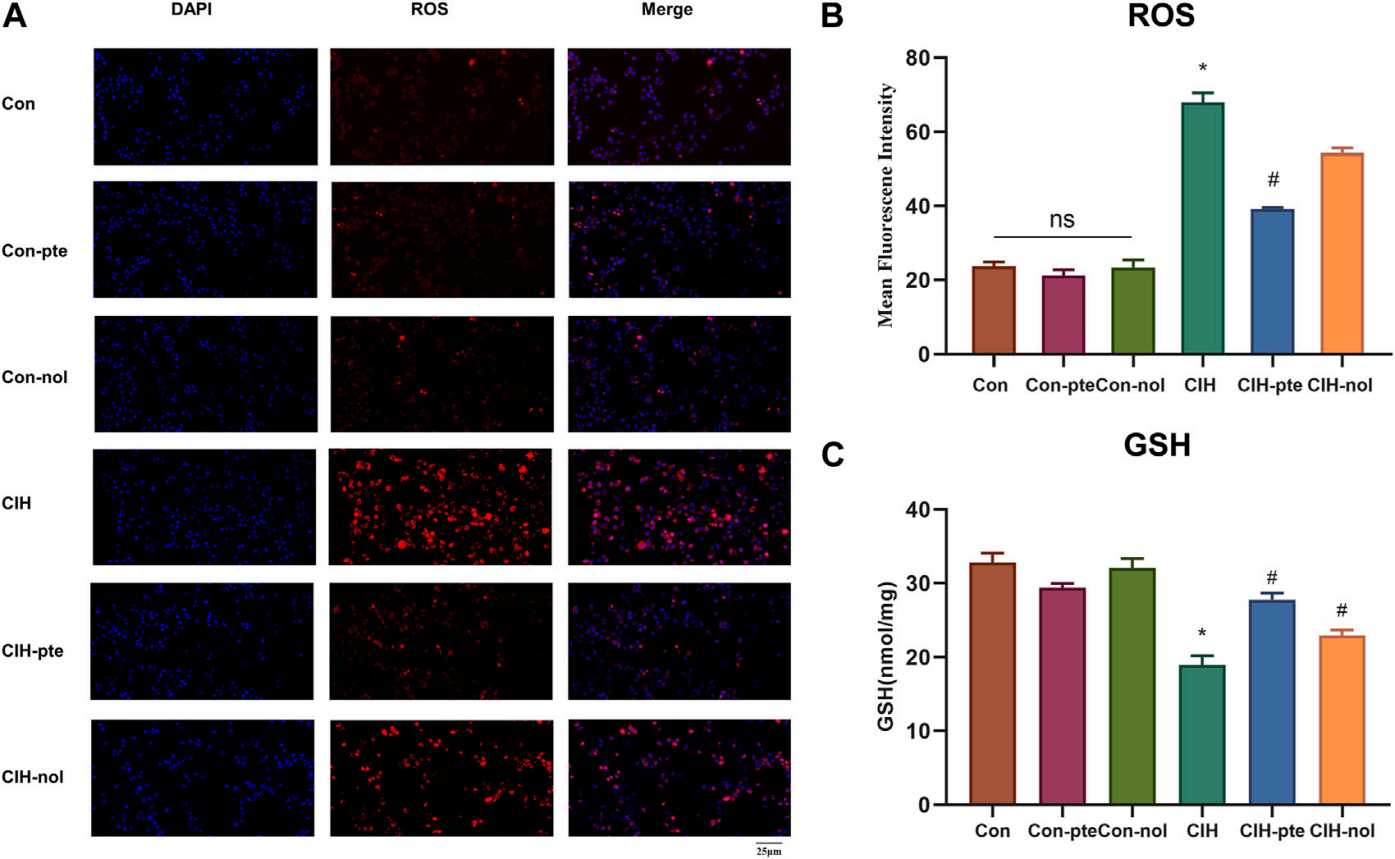
Pterostilbene and notopterol ameliorated the oxidative stress caused by CIH. **(A)** Red (ROS) and blue (DAPI) fluorescent signals. Original magnification is ×400. **(B)** Measurements of ROS in different groups (n = 3, per group). **(C)** GSH expression in cell supernatant. Data represent the means ± SE for three independent experiments. *p < 0.05 *versus* the Con group; ^#^ *p* < 0.05 *versus* the CIH group. Abbreviations: ROS, reactive oxygen species; DAPI,4′,6-diamidino-2-phenylindole,GSH,glutathione.

**FIGURE 8:**
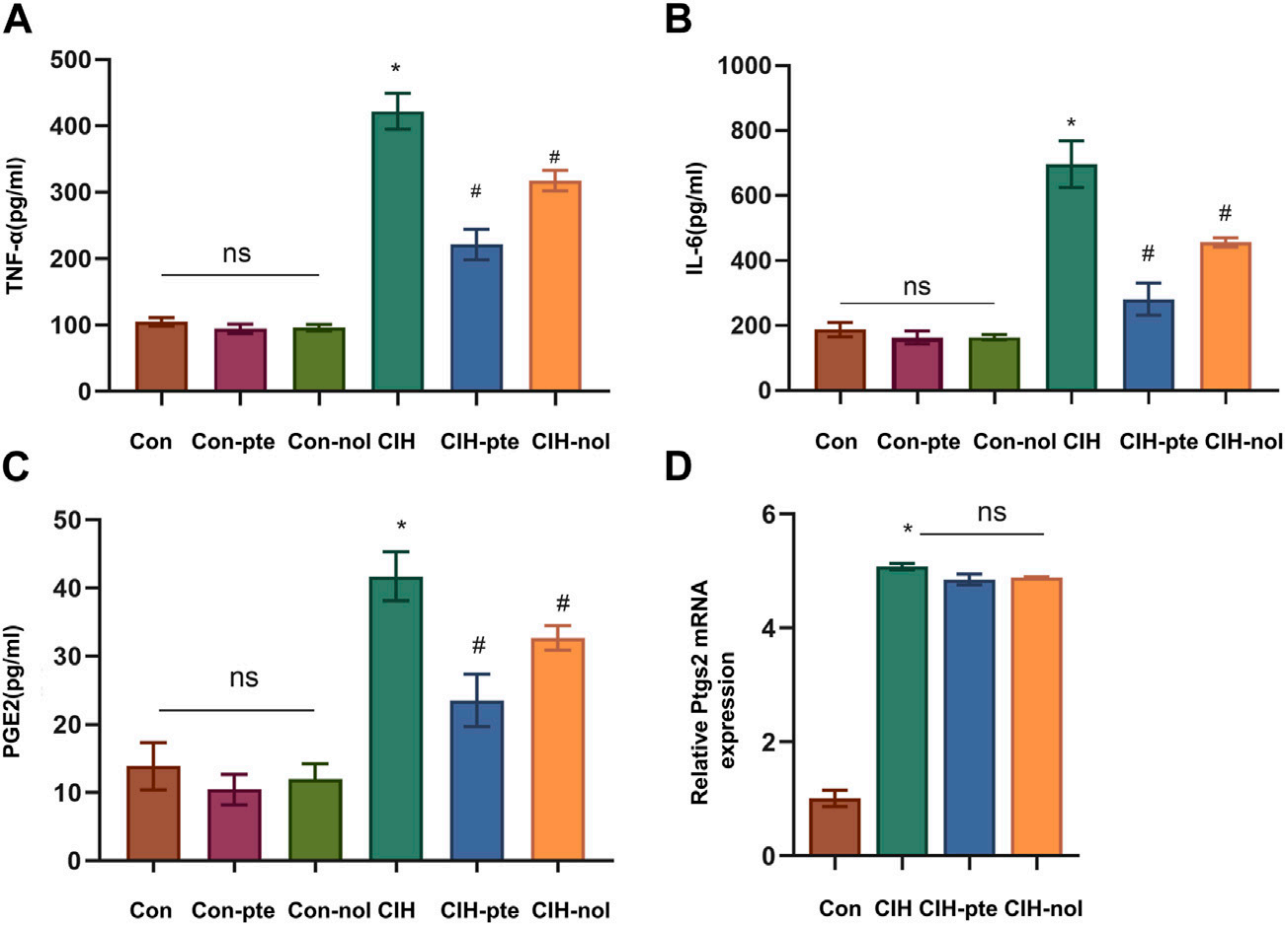
The inflammatory response produced by CIH is attenuated by pterostilbene and notopterol. **(A, B, C)** The enzyme-linked immunosorbent assay was used to measure the levels of TNF-α, IL-6, and PGE2 in the supernatants of HBE cells. **(D)** RT‒qPCR was used to quantify PTGS2 mRNA expression in different groups. Data represent the means ± SE for three independent experiments. *p < 0.05 *versus* the Con group;^#^ *p* < 0.05 *versus* the CIH group.

The results showed CIH increases ROS in HBE cells, while the addition of pterostilbene or notopterol reduced ROS, with the former being reduced to a greater extent (*p* < 0.05) (Figures 7A,B). GSH variations in various groups were inversely proportional to ROS increases (Figure 7C). The findings demonstrate that pterostilbene and notopterol can alleviate the oxidative stress caused by CIH. To investigate their impact on inflammation, we measured IL-6, TNF-α, and PGE2 levels by ELISA and found no differences between the Con, Con-pte, and Con-nol groups. IL-6, TNF-α, and PGE2 levels were dramatically elevated in the CIH group and significantly lowered in comparison to the control group after pretreatment with pterostilbene (20 µM) or notopterol (30 µM) for 6 h, although there was no significant difference between the two groups (Figures 8A–C). After CIH, the expression of PTGS2 mRNA in the cells greatly increased; however, there was no significant variance after adding pterostilbene and notopterol (Figure 8D).

### Pterostilbene and notopterol suppress the NF-κB pathway by decreasing PTGS2

**FIGURE 9:**
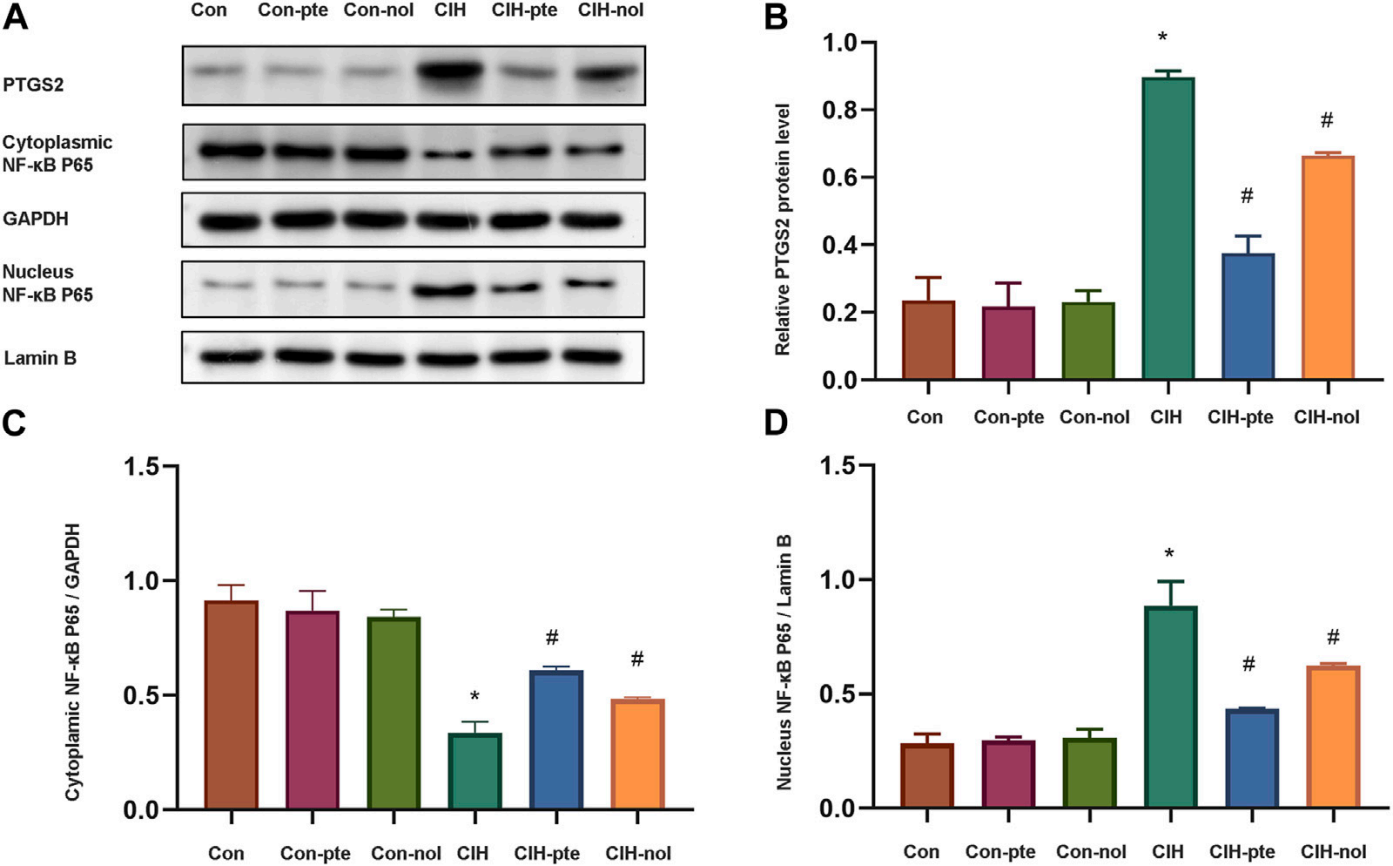
Pterostilbene and notopterol reduce PTGS2 by inhibiting the NF-κB pathway. **(A)** Western blotting was used to evaluate PTGS2, cytoplasmic NF-κB, and nuclear NF-κB. **(B)** The quantification of PTGS2 protein band densitometry. **(C) (D)** The quantifications of cytoplasmic NF-κB and nuclear NF-κB protein bands. Data represent the means ± SE for three independent experiments.*p < 0.05 *versus* the Con group;^#^ *p* < 0.05 *versus* the CIH group.

Further research on the mechanism of pterostilbene and notopterol in reducing inflammation and oxidation. The CIH group had significantly greater levels of PTGS2 than the normoxic group (Figures 9A,B). In the CIH group, a significant translocation of NF-κB from the cytoplasm to the nucleus was observed, indicating an alteration in its cellular distribution (Figures 9C,D). After 6 h of pretreatment with either pterostilbene (20 µM) or notopterol (30 µM), the levels of PTGS2 and NF-κB in the nucleus decreased significantly, with pterostilbene decreasing more than notopterol (Figure 9D). Our research demonstrates that both pterostilbene and notopterol suppressed the NF-κB pathway by decreasing PTGS2, with the former having a more significant effect.

### Pterostilbene increases PTGS2 ubiquitination

**FIGURE 10:**
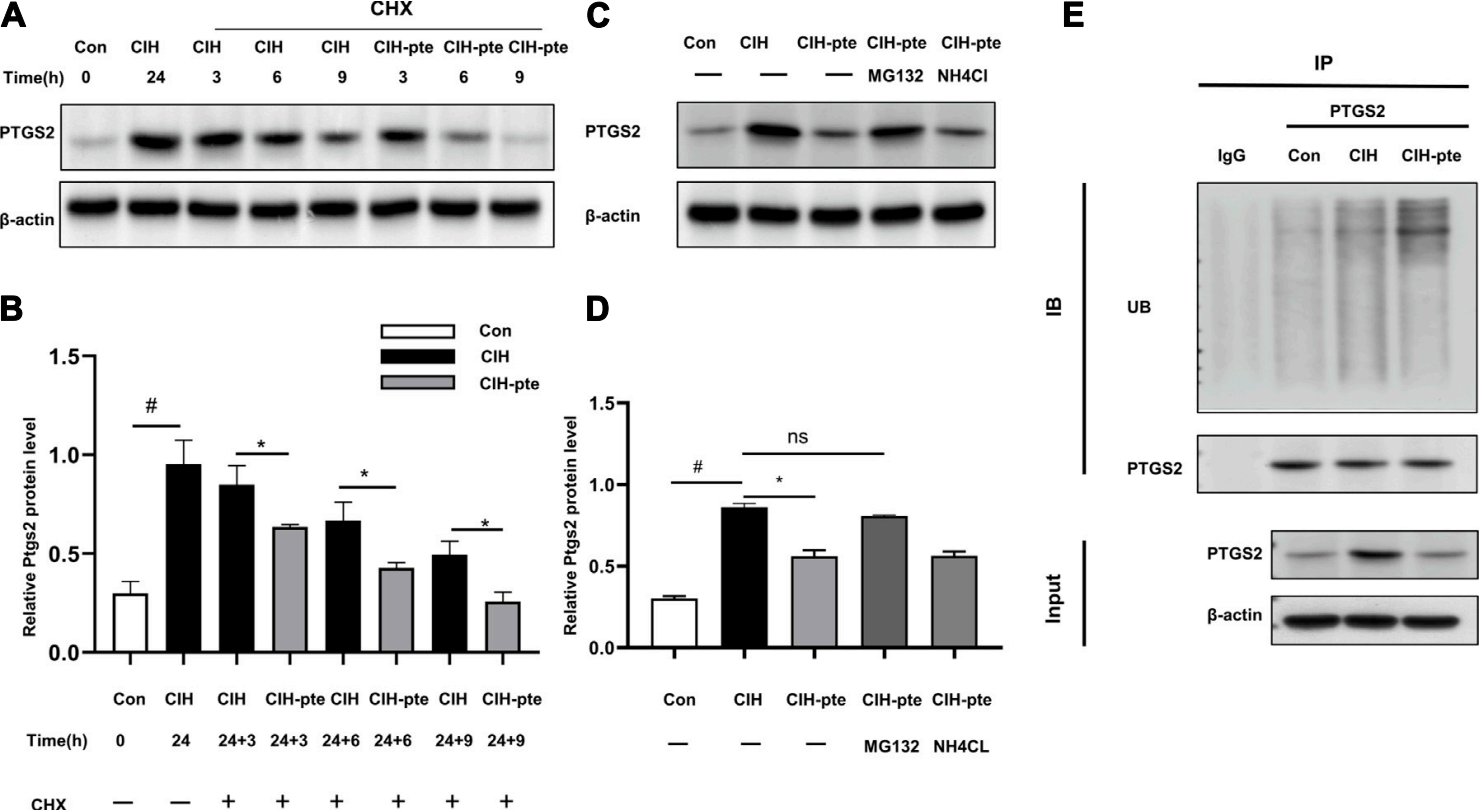
Pterostilbene enhances PTGS2 protein degradation by ubiquitination. **(A) (B)** Following the addition of cycloheximide, Western blotting was used to assess the quantity of PTGS2 protein in different groups, and quantitative analysis was performed. **(C, D)** Following the administration of MG132 or lNH4Cl, the protein concentration of PTGS2 was analysed by Western blotting, and quantitative analysis was performed. **(E)** The CoIP experiment revealed that the presence of pterostilbene promotes PTGS2 protein ubiquitination. Data represent the means ± SE for three independent experiments.*p < 0.05 *versus* the specified group,^#^ *p* < 0.05 *versus* the Con group.

We investigated the correlation between pterostilbene and PTGS2 in greater detail. The addition of the translation inhibitor cycloheximide (10 mM) to HBE cells after CIH(24 h) (24 h) resulted in progressive degradation of PTGS2 protein in all groups, although the degradation of PTGS2 was accelerated in the pterostilbene group compared to the control group (Figures 10A,B). This action was inhibited by MG132 at a dose of 20 µM but was unaffected by NH4Cl at a dose of 15 mM, indicating that the degradation of PTGS2 may accelerate the PTGS2 ubiquitination process (Figures 10C,D). The coIP experiments found that adding pterostilbene accelerated the formation of PTGS2 protein ubiquitination, increased the ubiquitin product, and accelerated degradation through the ubiquitin‒proteasome system (Figure 10E).

## Discussion

OSAS is defined as recurrent obstruction of the upper respiratory tract during sleep, which results in systemic intermittent hypoxia (Schwartz, 2009). Sleep deprivation, cardiovascular complications, metabolic disorders, and cognitive impairment are all associated with OSAS (Yerlikaya et al., 2018). Quintero, M illustrated that an increase in ROS causes CIH to become the pathophysiological mechanism of OSAS and that an aggravation of CIH can lead to an increase in oxidative stress and an inflammatory response (Quintero et al., 2013). These findings were supported by the observation that OSAS is associated with increased CIH.

The TCMSP database was used to examine 33 possible disease-related metabolites. The metabolites mostly worked on 50 genes, with PTSG2 being the most significant. Therefore, PTSG2 may play an important role in the pathogenesis of OSAS (Campillo et al., 2017). We chose two metabolites (pterostilbene and notopterol) for the experiment after reviewing the PubMed database and completing a literature search on hypoxia, oxidative stress, and inflammation (Tzeng et al., 2021). Prostaglandin-endoperoxide synthase (PTGS), also known as cyclooxygenase (COX), is a key enzyme in prostaglandin biosynthesis with dual dioxygenase and peroxidase activity (Li et al., 2018a). PTGS possesses two isoenzymes, constitutive PTGS1 and inducible PTGS2, which are regulated by unique stimuli, have distinct tissue distributions, and are regulated in different ways. They primarily involve inflammation and prostaglandin biosynthesis (Chulada et al., 2000). Numerous nonsteroidal anti-inflammatory medications target PTGS2, which is increased during inflammation; as a result, improper regulation of this gene is connected to the development of cancer and cardiovascular events (Ratliff, 2005). Pterostilbene, also known as 3′, 5′-deoxy-4-hydroxytoluene, has anti-inflammatory, antioxidant, and anti-apoptotic properties similar to resveratrol (Li et al., 2021). However, its antioxidant activity is greater than that of resveratrol, and it is believed to play an antioxidant, anti-inflammatory, blood glucose, and lipid regulation role within the human body (Li et al., 2018b; Kim et al., 2020). According to recent scientific research findings, it prevents the production of inflammatory factors and mitigates the harm lipopolysaccharide inflicts on the glial cells of mice (Zhu et al., 2020).

KEGG pathway analysis was performed on 50 OSAS genes associated with *Notopterygium incisum*. Among the 39 identified pathways, HIF-1A, PI3K-Akt, NF-κB, and apoptosis were most closely associated with inflammation and oxidative stress. Our previous research indicated that CIH can exacerbate bleomycin-induced interstitial pulmonary fibrosis in mice via the ROS/HIF-1 pathway and that HIF-1 plays a crucial role in CIH (Xiong et al., 2021). NF-κB is a nuclear transcription factor found in the cytoplasm that frequently occurs in an inactive form as a homologous or heterodimer (Liao et al., 2020). As a critical correlation molecule, NF-κB plays a crucial role in the inflammatory response, uncontrolled inflammation, and tumor formation, particularly when a large number of genes are activated in response to infection and inflammation (Zinatizadeh et al., 2021). A complex network governs NF-κB activity, and its molecular regulatory mechanism after activation remains unknown (Chen et al., 2021b). Hypoxia that occurs on a chronic and intermittent basis is one of the hallmarks of OSAS, which can stimulate the brain’s respiratory center (Lévy et al., 2015). In addition, CIH is reported to activate oxidative stress and ROS while simultaneously reducing SOD and GSH levels (Liu et al., 2018).

HBE cells were exposed to intermittent hypoxia in a hypoxic incubator for 24 h, first at 5% O_2_ for 5 min and then at 21% O_2_ for 10 min (Azietaku et al., 2017). There are multiple types of CIH cell models. Cells were exposed to 1.5% O_2_ for 30 s, followed by 20% O_2_ at 37°C for 5 min in three gas chambers (N_2_, O_2_, CO_2_) (Wang et al., 2020a). According to the findings of our study, the levels of IL-6, TNF-α, and PGE2 did not significantly change between the normoxic group, the normoxic plus pterostilbene group, and the normoxic plus notopterol group. However, they were considerably greater in the CIH group than in the normoxic group. This theory suggests CIH can stimulate cells to produce abundant inflammatory cytokines (Miao et al., 2020). OSAS can elevate inflammatory cytokine expression and decrease mucociliary transport in the upper airway, resulting in airway inflammation (In et al., 2021). Notopterol (30 µM) and pterostilbene (20 µM) did not produce significant cell damage in normal oxygenated cells, indicating that neither medication caused significant harm to HBE cells. CIH is an inducer of the inflammatory response (Timon et al., 2021). The results showed that pterostilbene or notopterol could reduce the inflammatory response of HBE cells to hypoxia. Pterostilbene has been found to minimize astrocyte inflammation in the brain, protect brain function, activate the Nrf2 pathway to regulate chondrocyte inflammation, ROS generation, and oxidative stress, and activate the Nrf2 pathway to protect against oxidative stress (Zhu et al., 2020). The lung is the primary organ affected by hypoxia in OSAS, and it was the lung that felt the shift in hypoxia first (Timon et al., 2021). We discovered that CIH induced an inflammatory response in HBE cells.

Through network analysis, we identified that the primary target of disease-related metabolites of *Notopterygium incisum* is PTGS2 and that the NF-κB pathway is its KEGG enrichment pathway. Compared to the control group, the CIH group showed a substantial increase in PTGS2 expression. The presence of CIH could cause HBE cells to produce an increase in PTGS2, which would then activate NF-κB and cause inflammation. Western blot analysis revealed that CIH could induce HBE cells to produce more PTGS2, which could activate the NF-κB pathway and promote the initiation of inflammation. In *Mus musculus*, targeted knockdown of PTGS2 expression inhibits the NF-κB signaling pathway, reduces the apoptosis of endothelial progenitor cells, and enhances the proliferation, migration, and angiogenesis of EPCs (Zhou et al., 2019). Through the PTGS2/NF-κB pathway, Curcuma longa and Allium both provide anti-inflammatory effects that are complementary to one another (Lee et al., 2020).

PTGS2 is activated and elevated in tumor tissues, and antitumour medications attempt to inhibit it through the Ub system. When cycloheximide was added to HeLa cells, parecoxib increased the degradation of the PTGS2 protein via the ubiquitin‒proteasome pathway (Neuss et al., 2007). Baicalin can induce PTGS2 protein ubiquitin degradation by inhibiting the HSP90/PTGS2 interaction (Zhang et al., 2022), which is consistent with our observation that pterostilbene reduces PTGS2 protein through the ubiquitin pathway. The degradation of PTGS2 protein in HBE cells was inhibited by the addition of the proteasome inhibitor MG132 but not the lysosomal inhibitor NH4Cl. The observation that PTGS2 protein was found in the input groups is evidence that the CoIP experiment was conducted correctly. The PTGS2 protein pull-down protein binding products contain ubiquitinated proteins, and pterostilbene can accelerate the rate of ubiquitination.

Pterostilbene may activate the proteasomal pathway, accelerate ubiquitination, and subsequently promote the degradation of PTGS2 molecules. Nonetheless, our findings were restricted to the *in vitro* experiment, the mechanism of the inflammatory response was difficult to comprehend, and many processes remained unknown.

## Conclusion

**FIGURE 11:**
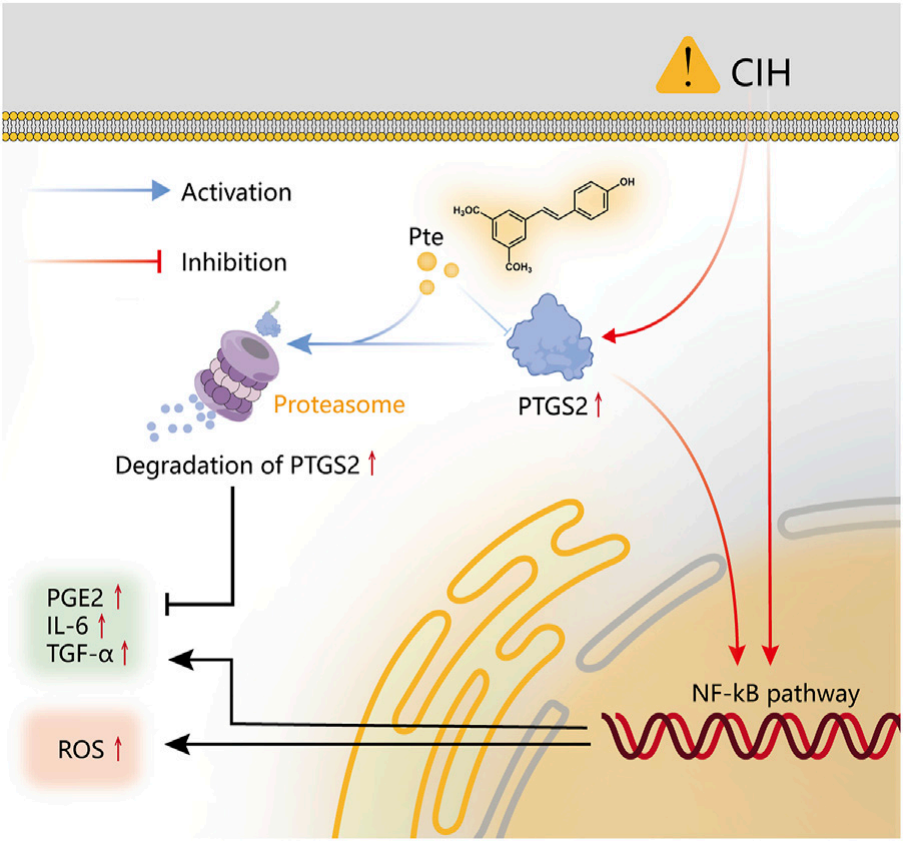
Schematic representation of the molecular process and mechanism.

We discovered that pterostilbene, one of the active metabolites in *Notopterygium incisum*, can reduce the inflammatory response produced by OSAS and CIH through network analysis and *in vitro* validation studies. Pterostilbene inhibits the release of inflammatory factors via the PTGS2/NF-κB pathway, whereas OSAS is associated with systemic inflammation and oxidative stress (Figure 11). This research can serve as a foundation for future investigations of the *in vivo* mechanism of OSAS.

## Data Availability

The datasets presented in this study can be found in online repositories. The names of the repository/repositories and accession number(s) can be found in the article/Supplementary Material.
