# Supplementary material for: Active metabolites and potential mechanisms of Notopterygium incisum against obstructive sleep apnea Syndrome (OSAS): network analysis and experimental assessment
Source: Front Pharmacol. 2023 Aug 31;14:1185100. doi: 10.3389/fphar.2023.1185100 (PMC10500596; doi:10.3389/fphar.2023.1185100)
Supplement: Supplementary file 1 [file Table1.docx]

**Table S1|** GO analysis of core targets(BP,CC,MF,top 9 pathways)

| **ID** | **Description** | **geneID** | **qvalue** |
| --- | --- | --- | --- |
| BP | response to oxidative stress | PTGS2/FOS/COL1A1/ALAD/RELA/IL10/BCL2/CASP3/DUOX2/NCF1/SP1 | 2.07E-06 |
| BP | response to oxygen levels | PTGS2/DPP4/SLC6A4/CCNA2/NOS2/COL1A1/ALAD/BCL2/CASP3 | 1.74E-05 |
| BP | response to hypoxia | PTGS2/DPP4/SLC6A4/CCNA2/NOS2/ALAD/BCL2/CASP3 | 6.10E-05 |
| CC | mitochondrial outer membrane | MAOB/PGR/HK1/BCL2/BAX/CASP8 | 0.000255086 |
| CC | NADPH oxidase complex | DUOX2/NCF1 | 0.004073156 |
| CC | ER to Golgi transport vesicle membrane | SREBF2/SREBF1 | 0.039598936 |
| MF | metal ion transmembrane transporter activity | SLC6A2/SLC6A4/KCNH2/SLC6A3/SCN5A/OPRM1 | 0.003211006 |
| MF | cytokine receptor binding | IL10/IL6R/CASP3/CASP8 | 0.011323673 |
| MF | oxidoreductase activity acting on NADPH | DUOX2/NCF1 | 0.002580843 |
