# Supplementary material for: Active metabolites and potential mechanisms of Notopterygium incisum against obstructive sleep apnea Syndrome (OSAS): network analysis and experimental assessment
Source: Front Pharmacol. 2023 Aug 31;14:1185100. doi: 10.3389/fphar.2023.1185100 (PMC10500596; doi:10.3389/fphar.2023.1185100)
Supplement: Supplementary file 2 [file Table2.docx]

**Table S2|** KEGG analysis of core targets(top10 pathways)

| **ID** | **Description** | **geneID** | **qvalue** |
| --- | --- | --- | --- |
| hsa04215 | Apoptosis - multiple species | BCL2/BAX/CASP9/CASP3/CASP8 | 1.81E-05 |
| hsa04210 | Apoptosis | FOS/RELA/BCL2/BAX/CASP9/CASP3/CASP8 | 9.19E-05 |
| hsa04066 | HIF-1 signaling pathway | NOS2/HK1/RELA/IL6R/BCL2/PRKCA | 0.00020352 |
| hsa04115 | p53 signaling pathway | BCL2/BAX/CASP9/CASP3/CASP8 | 0.00029956 |
| hsa04064 | NF-kappa B signaling pathway | PTGS2/RELA/BCL2/ERC1/ CXCL8 | 0.00284370 |
| hsa04151 | PI3K-Akt signaling pathway | COL1A1/G6PC3/RELA/IL6R/BCL2/CASP9/PRKCA | 0.00781274 |
| hsa04380 | Osteoclast differentiation | FOS/PPARG/RELA/NCF1 | 0.01153066 |
| hsa05012 | Parkinson disease | MAOB/SLC6A3/BAX/CASP9/CASP3 | 0.02523971 |
| hsa05010 | Alzheimer disease | PTGS2/NOS2/RELA/CASP9/CASP3/CASP8 | 0.02774066 |
| hsa04660 | T cell receptor signaling pathway | FOS/RELA/IL10 | 0.02843703 |
